# LC-MS/MS Method Minimizing Matrix Effect for the Analysis of Bifenthrin and Butachlor in Chinese Chives and Its Application for Residual Study

**DOI:** 10.3390/foods12081683

**Published:** 2023-04-18

**Authors:** So-Hee Kim, Yoon-Hee Lee, Mun-Ju Jeong, Da-Yeong Gwon, Ji-Ho Lee, Yongho Shin, Hoon Choi

**Affiliations:** 1 Department of Applied Bioscience, Dong-A University, Busan 49315, Republic of Korea; 2 Department of Life & Environmental Sciences, Wonkwang University, Iksan 54538, Republic of Korea; 3 Department of Crop Sciences, Konkuk University, Seoul 05029, Republic of Korea

**Keywords:** LC–MS/MS, matrix effect, Chinese chives, bifenthrin, butachlor, QuEChERS

## Abstract

The matrix effect refers to the change in the analytical signal caused by the matrix in which the sample is contained, as well as the impurities that are co-eluted with the target analyte. In crop sample analysis using LC–MS/MS, the matrix effect can affect the quantification results. Chinese chives are likely to exhibit a strong matrix effect when co-extracted with bifenthrin and butachlor due to the presence of phytochemicals and chlorophyll. A novel analytical method was developed to reduce the matrix effects of bifenthrin and butachlor to a negligible level in Chinese chives. The established method had a limit of quantitation of 0.005 mg/kg and correlation coefficients greater than 0.999 within the range of 0.005–0.5 mg/kg. Matrix effects were found to be negligible, with values ranging from −18.8% to 7.2% in four different sources of chives and two leafy vegetables. Compared to conventional analytical methods for the LOQ and matrix effect, the established method demonstrated improved performances. The analytical method was further applied in a residual study in chive fields. The active ingredient of butachlor 5 granule (GR) was not detected after soil admixture application, while that of bifenthrin 1 emulsifiable concentrate (EC) showed a range from 1.002 to 0.087 mg/kg after foliar spraying. The dissipation rate constant (*k*) of bifenthrin was determined to be 0.115, thus its half-life was calculated to be 6.0 days. From the results, PHI and safety use standards of both pesticides were suggested. The developed analytical method can be applied to accurately determine bifenthrin and butachlor residues in Chinese chives and provides a foundation for further research on the fate and behavior of these pesticides in the environment.

## 1. Introduction

In chromatography coupled with mass spectrometry (MS), the matrix effect is considered a challenging issue. The matrix effect refers to a phenomenon in which the quantitative results are altered due to signal suppression or enhancement [1]. The main causes of the matrix effect are the presence of matrices or other interferences in the sample extract. These impurities affect the ionization efficiency of electrospray ionization (ESI) and atmospheric pressure chemical ionization (APCI) in liquid chromatography (LC)-based MS by co-eluting with analytes [1,2]. In the field of gas chromatography (GC), it is well-known that the presence of matrices can impact the transfer of analytes from the GC injector to the column [3,4]. Strong matrix effects could lead to significant quantitation distortions.

Samples that have a higher dry matter content to total mass ratio usually produce a severe matrix effect. Samples with high water content, such as urine or saliva, tend to have a mild or negligible matrix effect, while samples with high levels of protein or fat, such as hair or livestock products, usually exhibit a strong matrix effect [5,6,7,8,9]. The matrix effect for crops can vary depending on their endogenous composition and biochemical characteristics. This variation can be attributed to the presence of various natural compounds, such as pigments, sugars, enzymes, proteins, lipids, and minerals in the crops.

If the matrix effect is significant in the sample, determining the residue levels using a typical standard calibration curve will result in inaccurate quantification outcomes. Several solutions exist to address this problem. One of them is the use of an internal standard (IS) that exhibits a similar matrix effect as the target analyte, allowing for the correction of the quantitation results by an internal standard calibration method. In mass spectrometry, an isotope-labeled IS is considered ideal. However, these materials are expensive and not available for multiresidue analysis, as each analyte exhibits a different matrix effect due to its distinct retention time [1].

Another alternative is using a matrix-matched standard [10]. It can be prepared by mixing the blank matrix solution from the sample extract and the standard solution. It can provide accurate results for multi-residue analysis because each analyte in an unknown sample will be subject to the same matrix effects as the corresponding analyte in the matrix-matched standard. However, it is not easy to find the same blank samples in large-scale monitoring. Even for the same crop, the matrices may vary depending on the cultivars, resulting in different matrix effects [11].

The most orthodox but ideal method is to reduce the matrix effect by removing as many impurities as possible during sample preparation. In LC-based MS, the ionization efficiency of analytes can be restored by removing their chromatographic co-eluting compounds (co-extracts). For leafy vegetables that are known to have high levels of pigments and phytochemicals, simple extraction and partitioning procedures may not be effective in removing most of the matrices. In such cases, incorporating additional purification methods, such as solid-phase extraction (SPE) or dispersive-SPE (d-SPE) can strongly remove impurities through physical-chemical properties, such as adsorption or ion exchange [12,13]. From this, high-quality results in quantitative determination can be achieved.

Chinese chives (*Allium tuberosum* Rottler), also known as gallic chives, are a perennial herb that belong to the genus Allium, the same genus as onions and garlic. They are widely cultivated and used in many Asian countries, especially China and Korea, where they are considered an important crop and are commonly used in traditional dishes, including soups, stews, and fermented foods, such as kimchi. They have also been traditionally used as herbal medicine and health supplements, and they have been reported to have a variety of medicinal properties. Studies have reported that chives have antioxidant [14,15] and anti-cancer effects [16]. Chives were also reported to have cholesterol-lowering effects and may have potential in preventing certain types of cardiovascular diseases [17,18]. Chives contain various phytochemicals that can aid in reducing symptoms of various diseases and in enhancing physiological activities [19,20]. However these compounds can also cause strong matrix effects in addition to the chlorophyll pigments present in chives. It is known that these can be efficiently eliminated by a primary secondary amine (PSA), graphitized carbon black (GCB) sorbents, or by hydrophilic-lipophilic balance (HLB) [21,22,23].

Bifenthrin is an insecticide that belongs to the pyrethroids. It is effective against a wide range of pests that feed on foliage, including beetles, flies, bugs, cicadas, moths, and grasshoppers, as well as some species of mites [24]. Bifenthrin affects the nervous system of insects by disrupting the function of neurons through interaction with sodium channels [25]. Butachlor is a selective systemic herbicide that is used for pre-emergence control of annual grasses and some broad-leaved weeds in both seeded and transplanted rice [24]. Butachlor is a chloroacetamide, and its mode of action is by blocking the elongase, which is responsible for the elongation of very long-chain fatty acids, as well as by inhibiting the enzymes involved in geranylgeranyl pyrophosphate cyclization [26,27].

These two compounds are classified as non-polar molecules with log *P* values > 6.0 and 4.5, respectively [24,28]. In terms of methodology, there is a high likelihood of strong matrix effects when the analytes are co-eluted with non-polar chlorophylls of chives. Their commercial products have been widely used and have established maximum residue limits (MRLs) for Chinese chives in the Republic of Korea. However, there are no “pre-harvest intervals (PHIs)” and “safety use standards” for bifenthrin emulsifiable concentrate (EC) and butachlor granule (GR) [29]. PHI is a period between the last pesticide application and the first day of crop harvesting. During this period, the pesticide concentration should decrease to a level below the MRL [30]. The safety use standard generally refers to the guidelines for the safe and proper use of pesticides, in which the PHI and the maximum number of pesticide applications are included. To determine PHIs and safety use standards, it is essential to understand the residue characteristics of pesticides on crops. Dissipation of pesticides can occur due to various reasons, such as degradation caused by rainfall and sunlight, and dilution of pesticide concentration as the crop grows [31].

When using conventional methods to determine pesticides quantitatively, the presence of phytochemicals and chlorophyll in chives is expected to cause a considerably stronger matrix effect for bifenthrin and butachlor. This is because these components are not properly eliminated during conventional sample preparation as well as co-extracted with the target pesticides. Therefore, it is essential to develop an alternative analytical method capable of reducing the matrix effect to a negligible level. In this study, various analytical methods were compared and the optimal analytical condition was found for bifenthrin and butachlor in chives, focusing on minimizing the matrix effect in LC–tandem MS (LC–MS/MS). The established method was evaluated for the matrix effects with target pesticides in various chive cultivars and other vegetables to verify their methodological ruggedness, and their analytical characteristics were also compared to those of the conventional methods. To establish PHIs and safety use standards of bifenthrin 1 (%) EC and butachlor 5 (%) GR, residue studies were conducted in two chive fields and the active ingredients were quantitively determined using the established method. By removing the variables from the matrix effect, the quantitative residual results are highly reliable, and the residue results can be considered reference data for constructing the safety use standards and PHI of pesticide products in chives.

## 2. Materials and Methods

### 2.1. Chemicals and Reagents

Bifenthrin (purity; 99.0%), butachlor (95.9%) standards, and ammonium formate (10 M) were purchased from Sigma-Aldrich (St. Louis, MO, USA). Commercial pesticide products were Talstar [bifenthrin 1 EC (NongHyup Chemical Co., Ltd., Seongnam, Korea)] and Macet [butachlor 5 GR (FarmHannong Co., Ltd., Seoul, Korea)]. HPLC grade acetonitrile (MeCN) and ethyl acetate (EA) were obtained from Samchun Chemical (Seoul, Korea) and from Duksan Pure Chemical (Seoul, Korea), respectively. LC–MS grade methanol (MeOH) and formic acid (>99%) were purchased from Thermo Fisher Scientific (Waltham, MA, USA). LC–MS grade water was obtained from Merck (Darmstadt, Germany). Acetic acid (99.5%) was purchased from Junsei Chemical Co., Ltd. (Tokyo, Japan). QuEChERS extraction original kit (4 g of magnesium sulfate; MgSO_4_ and 1g of sodium chloride; NaCl), EN 15662 kit (4 g of MgSO_4_, 1 g of NaCl, 1 g of sodium citrate; Na_3_Citr·2H_2_O, and 0.5 g of sodium hydrogen citrate sesquihydrate; Na_2_HCitr·1.5H_2_O), d-SPE, PSA (25 mg of PSA and 150 mg of MgSO_4_), and GCB (25 mg of PSA, 7.5 mg of bulk carbonate, and 150 mg of MgSO_4_) were obtained from Agilent Technologies (Santa Clara, CA, USA). Oasis PRiME HLB cartridge plus light (100 mg) was purchased from Waters Corporation (Milford, MA, USA).

### 2.2. Standard Solutions and Calibration Curves

The bifenthrin and butachlor standards were precisely weighed considering their purity. The stock solutions (1000 mg/L) were prepared by dissolving each standard with MeCN. They were then diluted with MeCN to prepare a 10 mL working solution with a concentration of 20 mg/L, and subjected to serial dilution to 0.5, 0.25, 0.1, 0.05, 0.025, 0.01, and 0.005 mg/L. To plot a calibration curve, 300 μL of individual working solution and 300 μL of chive control extracts were mixed to prepare matrix-matched standard solutions to obtain 0.25, 0.125, 0.05, 0.025, 0.0125, 0.005, and 0.0025 mg/L. Five microliters of matrix-matched standard solutions were injected into LC–MS/MS. The correlation between the peak areas and the concentrations on the chromatogram was confirmed and the calibration curve was plotted.

### 2.3. LC–MS/MS Analytical Conditions

The analysis was carried out on a Shimadzu LCMS-8040 triple quadrupole mass spectrometer (Kyoto, Japan) coupled with Nexera liquid chromatograph (Shimadzu) consisting of a degasser (DGU-20A5), pump (LC-30AD), auto sampler (SIL-30AC), communications bus module (CBM-20A), and column oven (CTO-20A).

Chromatographic separation was performed on a Kinetex PS C18 column (2.6 μm, 3 × 100 mm; Phenomenex, Torrance, CA, USA). Mobile phase A consisted of ammonium formate (5 mM) and formic acid (0.1%) in water, and mobile phase B consisted of ammonium formate (5 mM) and formic acid (0.1%) in methanol. The gradient of the mobile phase B was started at 30% (0.0–0.2 min), increased to 90% (0.2–0.5 min), ramped to 98% (0.05–6 min), and then held (6–9 min). Then, it decreased to 30% (9–9.1 min) and equilibrated (9.1–13 min) as the initial percent ratio. The column oven temperature, flow rate, and injection volume were 40 °C, 0.2 mL/min, and 5 μL, respectively.

For the MS conditions, target pesticides were analyzed with positive electrospray ionization (ESI+) and multiple reaction monitoring (MRM) modes. The argon (99.999%) was selected as the collision-induced dissociation (CID) gas. The temperatures of the desolvation line (DL) and heat block were 250, and 400 °C, and the flow rates of the drying gas and nebulizing gas were 15 and 3 L/min, respectively. Data processing was performed using Shimadzu LabSolutions software version 5.60 SP2.

For qualitative and quantitative analyses, mass to charge ratio (*m*/*z*) ranging from 150 to 800 was scanned at 410 u/s in a full scan mode, and each precursor ion of a pesticide was selected. Fragmentation of the precursor ion was verified through a product ion scan under various collision energies (CEs), and quantifier and qualifier ions of MRM transitions and their CE were optimized based on the sensitivity and selectivity.

### 2.4. Comparison of the Sample Extraction and Partitioning

In order to optimize the extraction and partitioning conditions, several types of methods were compared. The extraction was conducted using MeCN, EA, and a mixture of MeCN and EA (1:1, *v*/*v*). The extraction samples were then partitioned with QuEChERS original (unbuffered) and EN 15662 (citrate-buffer) kit. Without purification procedures, each extraction-partitioning method was cross-compared using recovery and matrix effect studies. In the recovery study, 0.1 mL of standard solution at 10 mg/L was spiked to 10 g of the control chive samples to give a concentration in the sample of 0.1 mg/kg (*n* = 3). The sample was prepared using each procedure, and 5 µL of the extracts was injected into LC–MS/MS. A recovery rate was obtained by comparing signals of recovery samples and matrix-matched standard solutions. In the investigation of the matrix effect, a comparison was made between the chromatographic area of analytes in the matrix-matched standard solution and the pure standard solution (*n* = 3).

### 2.5. Comparison of the Sample Purification

Three kinds of procedures using PSA d-SPE, PSA + GCB d-SPE, and Oasis PRiME HLB SPE were compared for the optimization of purification. Samples that went through the extraction and partitioning procedures of Section 2.4 were pretreated under each purification condition, and their recoveries, matrix effects, and colorimetric analysis were compared.

### 2.6. The Established Method

Upon weighing 10 g of the homogenized samples into a 50-mL conical tube, 10 mL of MeCN was added. The sample was extracted for 2 min at 1300 rpm with a Genogrinder (1600 Mini-G, SPEX SamplePrep, Metuchen, NJ, USA) followed by adding 4 g of MgSO_4_ and 1 g of NaCl, shaken for 1 min at 1300 rpm, and centrifuged for 5 min at 3500 rpm using a centrifuge (M15R, Hanil Scientific, Gimpo, Korea). One milliliter of supernatant was taken into a GCB dSPE tube containing 25 mg of PSA, 7.5 mg of bulk carbonate, and 150 mg of MgSO_4_. Following mixing for 1 min using a vortex mixer, the insoluble solid in the sample was precipitated by a microcentrifuge (1248, Labogene, Lillerød, Denmark) at 13,000 rpm for 5 min. The supernatant (300 μL) was taken into a 2-mL vial, mixed with 300 μL of MeCN for matrix-matching, and injected into LC–MS/MS by 5 μL.

### 2.7. Method Validation

The limit of detection (LOD) and limit of quantitation (LOQ) were determined as the lowest concentration of the analyte that resulted in signal-to-noise ratios (S/Ns) greater than 3 and 10, respectively. The linear regression equation of the calibration curve was used, and its linearity was evaluated using the correlation coefficient (*r*^2^).

The recovery study involved adding 0.1 mL of standard solutions with concentrations of 1 mg/L, 10 mg/L to 10 g of the control chives, resulting in residual amounts of 0.01 and 0.1 mg/kg, respectively (*n* = 3). The samples were prepared using the established method and analyzed by LC–MS/MS. In order to verify the quantitation properties for concentrations exceeding the linear range of calibration of bifenthrin, 0.1 mL of a standard solution at 200 mg/L was treated to 10 g of the control sample (*n* = 3). It was then prepared using the established method, and 60 μL of extract solution was diluted with 240 μL of blank matrix-matched standard solution and 300 μL MeCN. To evaluate storage stability at 0.1 mg/kg, the samples that were treated with bifenthrin were kept for 38 days, and the samples treated with butachlor were kept for 25 days at a temperature of −20 °C. Each test was evaluated using accuracy and precision by calculating the recovery rate of the corresponding concentration.

To verify the ruggedness of the matrix effect in this method, three different origins of chives from the Republic of Korea, one from China, and two other green vegetables (shallot and spinach) were studied. The crops were prepared according to the established method, and their matrix-matched standard calibration curves and pure standard solution curves were compared (*n* = 3). The percentages of the matrix effects (% ME) for each analyte were calculated according to the equation below.

(1) $$\%ME=\left(\frac{Slope\;of\;the\;matrix\;matched\;standard\;calibration\;curve}{Slope\;of\;the\;pure\;standard\;carlibration\;curve}-1\right)\times 100$$

### 2.8. Field Experiment and Residue Analysis

**Figure 1:**
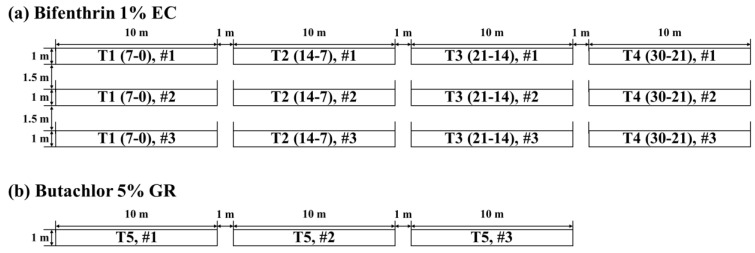
A design layout of the experimental fields in (**a**) Jeonju-si for bifenthrin 1 EC; (**b**) Jincheon-gun for butachlor 5 GR.

A residual study using the bifenthrin 1 EC was conducted in a greenhouse in Jeonju-si, Jeollabuk-do, Republic of Korea. The planting density was 15 × 15 cm. The study consisted of four treatment groups and one control group in the area of 10 m^2^ (1 × 10 m) for each group. According to the “pesticide safety use standards” for similar crops, the EC was diluted 1000-fold and sprayed at a dose of 150 L/10 a (0.0015 kg a.i./10 a) using a back-carried sprayer (MSB1500Li, Maruyama, Tokyo, Japan). The pesticide was sprayed twice, and the days of pesticide application before harvest for each treatment group (T) were as follows: 7 and 0 days (T1; 7-0); 14 and 7 days (T2; 14-7); 21 and 14 days (T3; 21-14); 30 and 21 days (T4; 30-21), [Figure 1a]. Buffer sections of 1 m^2^ (1 × 1 m) were placed to prevent cross-contamination between the experimental groups. A Thermo Recorder TR72wb thermo-hygrometer from T&D Corporation (Matsumoto, Japan) was installed and it measured the temperature and humidity in the field. Chive samples were harvested for each experimental group on 28 May 2022.

The other residual study using butachlor 5 GR was conducted in open-field in Jincheon-gun, Chungcheongbuk-do, Republic of Korea. The planting density was 20 × 20 cm. The study consisted of one treatment group (T5; *n* = 3) and one control group in the area of 10 m^2^ (1 × 10 m). The GR was treated through the soil admixture method before planting (11 April 2022) at a dose of 3 kg/10 a (0.15 kg a.i./10 a) [Figure 1b]. Chive samples were harvested three times on 9, 16, and 23 June 2022.

All samples were collected in amounts of more than 1 kg per each experimental group to ensure representativeness, transported to the laboratory, immediately homogenized, and stored at −20 °C until analysis.

### 2.9. Pesticide Residue Characteristics

For the bifenthrin 1 EC, the residual amount of each experimental group was determined to evaluate the dissipation patterns, dissipation rate constant, and biological half-life in days of the active ingredient. An equation for the first-order rate was used as follows:

(2) $$C_{t}=C_{0}e^{-kt}$$

where *C*_0_ is the concentration at 0 day, *k* is the dissipation rate constant, and *t* is days after pesticide spraying.

From the dissipation rate constant, a half-life in chives was calculated.

(3) $$t=\frac{ln2}{k}=\frac{0.693}{k}$$

## 3. Results and Discussion

### 3.1. The Established MRM Conditions

**Figure 2:**
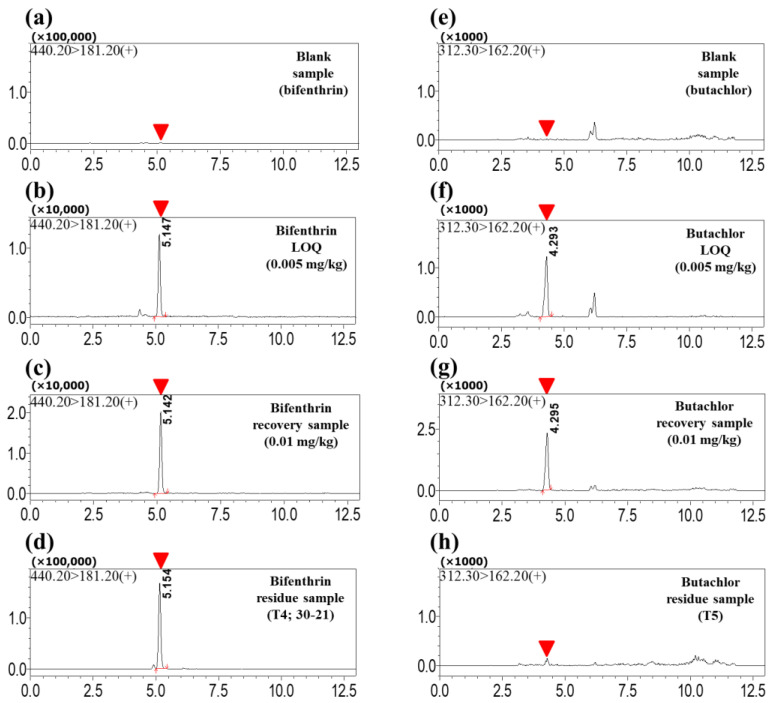
Chromatograms for bifenthrin and butachlor using the established MRM conditions; (**a**) blank samples without bifenthrin, (**b**) bifenthrin matrix-matched standard at LOQ (0.005 mg/kg), (**c**) bifenthrin recovery sample at 0.01 mg/kg, (**d**) bifenthrin residue sample (T4; 30-21), (**e**) blank samples without butachlor, (**f**) butachlor matrix-matched standard at LOQ (0.005 mg/kg), (**g**) butachlor recovery sample at 0.01 mg/kg, (**h**) butachlor real sample (T5).

The monoisotopic masses of bifenthrin and butachlor are 422.1 and 311.2 g/mol, respectively. Their *m*/*z* values in full scan mode were determined to be 440.2 and 312.3, indicating that they were ionized as [M + NH_4_]^+^ and [M + H]^+^, respectively. It is remarkable that the ionization pattern of bifenthrin differs from that of typical pesticides on LC–MS/MS. This ionization pattern has been observed in other LC-based mass spectrometry studies [32,33]. To improve the ionization efficiency of bifenthrin, it is necessary to add the ammonium formate that produces the ammonium (NH_4_^+^) adduct to the mobile phases. Detailed MRM conditions and retention times (t_R_) of target pesticides were described in Table 1. Figure 2 shows chromatograms for bifenthrin and butachlor using the established MRM conditions.

### 3.2. Optimization of the Sample Extraction and Partitioning

The preparation conditions that have been cross-compared using each extraction-partitioning method are as follows: MeCN + Original (M1); MeCN + EN 15662 (M2); MeCN/EA + Original (M3); MeCN/EA + EN 15662 (M4); EA + Original (M5); and EA + EN 15662 (C6). Table 2 illustrates the recovery and matrix effect results for bifenthrin and butachlor. In all combinations of extraction-partitioning procedures, the recovery rates were within 70–120%, satisfying the SANTE 11312/2021 guideline [34]. However, M5 and M6, where EA was used as the sole extracting solvent, showed larger relative standard deviations (RSDs) for the target analytes as well as lower recoveries for butachlor among the methods. Kecojeviiic et al. (2021) also demonstrated that extraction efficiency of MeCN was superior to that of EA [35].

The results for M1 to M4 indicate that the citrate buffer is not effective for these pesticides, as there was no significant difference in recovery between samples with or without the buffer. Rather, when the buffer was added under the same solvent conditions (M1 vs. M2; M3 vs. M4; M5 vs. M6), the relative standard deviation (RSD) tended to increase in most samples.

The matrix effect is typically classified as ’soft’ when it falls within −20% to 20%, ‘medium’ within −50% to −20% or 20% to 50%, and ‘strong’ when it falls below −50% or above 50% [36,37]. In this study, % ME was observed to be −50.5% to −20.4% in all methods, indicating medium to strong signal suppression (Table 2). As these methods did not include an appropriate purification process, co-extracts from the matrices resulted in notable matrix effects [38]. It was observed that increasing the proportion of EA under the same salt type conditions (M1 vs. M3 vs. M5 and M2 vs. M4 vs. M6) led to an increase in the RSD of both pesticides. Conversely, using the same solvent conditions (M1 vs. M2; M3 vs. M4; M5 vs. M6), the RSD of the majority of the samples decreased when the unbuffered salt was used.

In the multiresidue studies comparing the original and EN 15662 methods, a higher proportion of pesticides were found to show a soft effect range in the original method [39,40]. The addition of buffer components to the partitioning salts can improve the recovery of certain polar pesticides, but it could result in a negative impact on the matrix effect. The original method is still widely used because it is more rugged and less costly compared to buffer methods [41]. Based on the results of recovery and matrix effect, the M1 and M3 were selected as the optimal extraction-partitioning procedures. 

### 3.3. Optimization of Sample Purification

Without purification procedures, the signals of the pesticides were suppressed by the matrix effect (Table 2). These two compounds are non-polar pesticides, with log *P* values of >6.0 and 4.5, respectively [24,28]. Therefore, severe matrix effects can occur when they are co-eluted with non-polar pigments in LC–MS/MS. To address this issue, additional purification methods are required to remove these matrices. In this study, four types of purification methods were combined with M1 and M3 as follows: PSA d-SPE (A); PSA + GCB d-SPE (B); PRiME HLB (C); and PSA + GCB d-SPE followed by PRiME HLB (D). 

**Figure 3:**
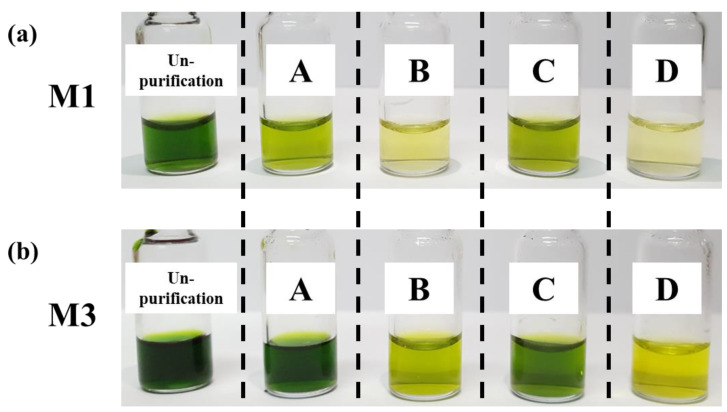
Colorimetric estimation of extract solutions for various purification methods. (**a**) Extraction with MeCN; (**b**) Extraction with MeCN/EA (1:1, *v*/*v*). The methods corresponding to capital letters A to D in this figure are as follows: A, PSA d-SPE; B, PSA + GCB d-SPE; C, HLB; D, PSA + GCB d-SPE and HLB combination.

One method for assessing the purification efficiency is through colorimetric estimation [23,42]. Under the same purification conditions, more green pigments were removed when MeCN was used as the extracting solvent (M1) compared to when MeCN/EA mixture (M3) was used (Figure 3). Under the same extraction conditions, purification methods, including GCB sorbents were the most effective in eliminating pigments. GCB effectively absorbs heavy pigments while not strongly bonding with pesticides, making it highly selective. GCB is known to effectively eliminate non-polar and planar molecules, such as chlorophylls, leading to a reduction in matrix effects through the removal of the co-extracts of non-polar pesticides [23,43,44]. Therefore, it is useful in leafy vegetables.

Table 3 shows the recovery and matrix effect results for bifenthrin and butachlor. In all methods, the recovery rates were within the range of 70–120% and RSDs were ≤20%, indicating that the purification process did not decrease the recoveries of target pesticides. It is noteworthy that the matrix effect of bifenthrin significantly decreased in all purification methods compared to the untreated preparation method (Table 3). Among them, the method using PSA dSPE under MeCN extraction (M1-A), and the method using PSA + GCB dSPE regardless of the extraction solvent used (M1-B, M3-B), showed a soft matrix effect of −9.0% to −3.2%. The soft effect (within ±20%) is considered to be a range in which the matrix effect can be negligible [45,46]. Therefore, we can conclude that these combinations effectively eliminated the co-extracts of bifenthrin within chives.

It was also confirmed that the use of PSA + GCB reduced the matrix effect of butachlor to the soft effect range”. This means that other co-extracts of butachlor, which have a different retention time than bifenthrin, are also well removed. However, when the same purification method was used together with PRiME HLB (M1-D and M3-D), there was no significant improvement in purification observed. Based on the studies of colorimetric estimation and the matrix effect, the M1-B combination was ultimately selected as the established preparation method.

### 3.4. Method Validation

In order to confirm the suitability and reliability of the established analytical method, validation, including the LOD, LOQ, linearity of calibration, recovery, storage stability, and matrix effect was conducted. For both bifenthrin and butachlor, a minimum concentration of 0.0015 mg/kg was determined as the LOD as it satisfied the definition of S/N being greater than 3, and the lowest concentration of 0.005 mg/kg satisfying S/N greater than 10 was chosen as the LOQ. The sensitivity of the analytical method was found to be superior to that of conventional methods used for analyzing bifenthrin and butachlor in leafy vegetables (LOQ range; 0.01–0.03 mg/kg) [47,48]. In the Republic of Korea, the MRLs established for chives are 0.5 mg/kg for bifenthrin and 0.05 mg/kg for butachlor, and that of bifenthrin is set only at 0.02 mg/kg in the EU [29,49]. For pesticides that are not included in the MRLs, the maximum tolerance concentration in crops is generally set at 0.01 mg/kg in many countries. Therefore, it can be concluded that the LOQ at 0.005 mg/kg in this method is adequate for detecting and quantifying target pesticides in chives. The calibration curve ranged from 0.0025 to 0.25 mg/L (corresponding to 0.005–0.5 mg per kg chives), and a weighting factor was not adopted. The study demonstrated good linearity with correlation coefficients (*r*^2^) of 0.9993 for bifenthrin and 0.9995 for butachlor.

The recovery study conducted at concentrations of 0.01 and 0.1 mg/kg showed acceptable averages of 101.3% and 99.5% for bifenthrin (RSD ≤ 5.9%), and 107.3% and 106.0% (RSD ≤ 2.9%) for butachlor, in accordance with the criteria (70–120% with RSD ≤ 20%) specified in the SANTE guideline (Table 4) [34]. For bifenthrin, the recovery at 2 mg/kg, which exceeded the highest calibration point, was further verified due to its expected presence as a residue. The dilution method did not compromise the accuracy of bifenthrin (88.0%). The storage stability study was conducted to check for changes, such as degradation or transformation of pesticides, that might occur during the storage of samples (Table 4). The results showed that the target pesticides remained stable in chives when stored at a frozen temperature of −20 °C, with recovery rates ranging from 104.3 to 109.6% (RSD ≤ 4.9%).

To test the method’s ruggedness, the matrix effects were evaluated using four types of chives collected from different sources or cultivation regions (Table 5). The C1 sample, which was used to develop the analytical method, had matrix effect values (% MEs) for the target pesticides ranging from −5.2% to −5.1% (RSD ≤ 4.4%). The Chinese chives (C4) purchased from the commercial market also showed a soft matrix effect of −0.1% to 3.0% (RSD ≤ 4.7%). The % MEs for the chives grown and harvested during this study were −1.2% to 1.8% (RSD ≤ 1.4%) for C2 and −18.8% to 2.1% (RSD ≤ 5.2%) for C3. The %Mes for both pesticides in all chive cultivars fell within the range of a negligible matrix effect, indicating that the analytical method is quantitatively rugged.

Using nonparametric statistics, the %ME of bifenthrin in C3 was found to be significantly lower than that in C2 (the Mann–Whitney U test, *P* < 0.05). These samples were from different cultivars (C2: Greenbelt and C3: Wangbelt) and grown in different regions, suggesting that the matrix effect may differ based on their variables, even for the same crop. Damale et al. (2023) reported the same suggestion for other crops [11]. Thus, it is crucial to eliminate as many matrices as possible during sample preparation. In our study, the matrices were efficiently removed through the use of PSA and GCB sorbents, resulting in a soft effect in all chive samples. This allowed for the assurance of ruggedness in the analytical method. To evaluate the versatility of this method, it was applied to other leafy vegetables (shallot and spinach) and showed a negligible matrix effect ranging from −11.0% to 7.2% (RSD ≤ 3.8%). Conventional QuEChERS methods showed a matrix effect of 70% in butachlor, which shows a stronger matrix effect than those of our study [47]. Therefore, the established method can be applicable for green vegetables, and it is possible to use the pure standard solutions for quantitation instead of the matrix-matched standard solutions [7,9].

### 3.5. Determination of the Pesticide Residues from the Field Study

To evaluate the residue patterns of butachlor and bifenthrin, the pesticides were sprayed in chive fields in Jincheon-gun and Jeonju-si, respectively. Neither of the compounds were found in the samples from the control plots. For the butachlor 5 GR, it was treated through the soil admixture method before planting, and the chive samples were harvested after 52, 59, and 66 days, which are the average growth periods for chives in Korea. The residue concentration of butachlor was below the LOQ (<0.005 mg/kg) in all samples. In another residue study, the butachlor 5 GR applied to paddy soil was not detected above the LOQ in the brown rice samples harvested after 122 days of application [50]. Although butachlor is a systemic herbicide [24], the rapid decomposition of butachlor by soil microorganisms prevents its uptake into crops [51]. As the residual amounts of butachlor in all chive samples were below the MRL, the guideline of “treatment with pesticide once to the soil” is determined to be an appropriate safety use standard.

For bifenthrin 1 EC, the residual amounts of the treatment groups sprayed on different days were compared (Table 6). The average residual concentrations for T1 to T4 ranged from 1.002 to 0.087 mg/kg, showing a steady decrease over time. The residual concentration in T1 was the highest, and it decreased by 73.8% between T1 and T2, dissipating rapidly within a week. The decrease in residual concentration in T3 and T4 compared to T1 was 87.6% and 91.3%, respectively. It was found that over 90% of the pesticide was dissipated within three weeks. According to Park et al. (2012), the active ingredient was found at a level of 0.74 mg/kg on day 0 in chives after the application of bifenthrin 1 WP [52], which is lower than that in our study. Additional information, such as weather and soil conditions is necessary to explain the acceptable reason for the difference in residual results.

**Figure 4:**
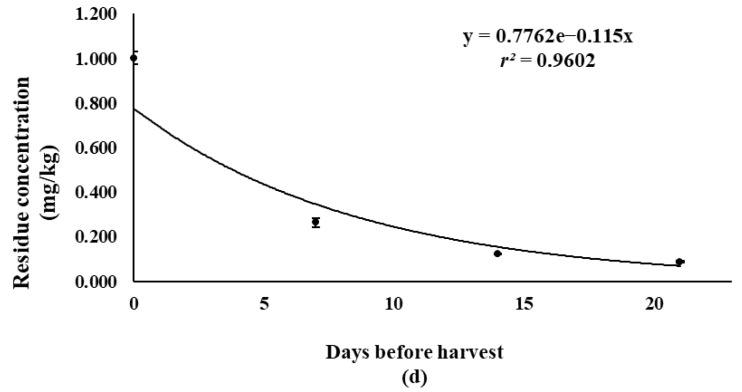
Dissipation patterns of bifenthrin residue in chives.

The dissipation patterns of bifenthrin 1 EC in chives were confirmed, and the dissipation rate constant (*k*) and half-life were calculated. It followed first-order kinetics reactions with the equation *y* = 0.7762e^−0.115*x*^ and an *r*^2^ of 0.9602, resulting in a *k* value of 0.115. (Figure 4). The half-life of bifenthrin was found to be 6.0 days in chives, whereas Park et al. (2012) reported a half-life of 3.8 days for bifenthrin 1 WP in chives [52]. In another study by Reddy et al. (2014), the half-life of bifenthrin 10 EC in cabbage was recorded as 2.7 days [53]. The differences in the half-life can result from various factors, such as the type of pesticide formulation, crop, field temperature and humidity, and metabolic activity [54]. Further studies are necessary under controlled conditions to determine the specific causes. The data on the dissipation rate constant and half-life data are crucial in establishing safety use standards and PHI for pesticides in crops [55]. All groups except for T1 had residual amounts below the MRL (0.5 mg/kg) based on the first-order kinetics reactions model. Therefore, it is determined to that it is appropriate to establish the safety use standard of up to two applications with the PHI of 7 days.

## 4. Conclusions

The study focused on the development and application of the LC–MS/MS method for the analysis of two pesticides, bifenthrin and butachlor, in Chinese chives. The d-SPE containing GCB sorbents was the most effective in eliminating co-extracts, thus resulting in a reduction of the matrix effect of the target analytes. The method was validated and the LOQ for both compounds was determined to be 0.005 mg/kg, which is adequate to cover the MRLs for chives in the Republic of Korea and the EU, and its sensitivity was better than that of conventional methods. The study found that the matrix effect of pesticides in four different sources of chives and two leafy vegetables (shallot and spinach) was negligible (% ME within ±20%), indicating that the method is rugged and versatile. The analytical method was applied in the residue study to assess the residue patterns of bifenthrin 1 EC and butachlor 5 GR in chive fields in Korea. Butachlor was not detected above the LOQ after soil admixture application, thus the guideline, “treatment with pesticide once to the soil”, was recommended as the safety use standard. For bifenthrin, the residual concentration declined rapidly over time after foliar spraying and followed first-order kinetics reactions, with a half-life of 6 days. From the results, the guideline “PHI 7 days and spraying no more than twice before harvesting”, was recommended as the safety use standard.

## Figures and Tables

**Table 1:** Retention times (t_R_), MRM conditions, and their collision energies (CEs) of bifenthrin and butachlor used for LC–MS/MS.

Pesticide	t_R_ (min)	Monoisotopic Mass	Ionization Type of Precursor Ion	Precursor Ion > Product Ion (CE, V)	
				Quantifier	Qualifier
Bifenthrin	5.1	422.1	[M + NH_4_]^+^	440.2 > 181.2 (−15)	440.2 > 166.1 (−41)
Butachlor	4.2	311.2	[M + H]^+^	312.3 > 162.2 (−21)	312.3 > 147.2 (−35)

**Table 2:** Recovery rates and matrix effects (MEs) of bifenthrin and butachlor at 0.1 mg/kg fortification according to various extraction solvents and partitioning salt types.

Method	Extraction Solvent	Partitoning Salt Type	Recovery (*n* = 3)				ME (*n* = 3)			
			Bifenthrin		Butachlor		Bifenthrin		Butachlor	
			Value (%)	RSD (%)	Value (%)	RSD (%)	Value (%)	RSD (%)	Value (%)	RSD (%)
M1	MeCN	Unbuffered ^1^	93.7	4.7	100.0	0.2	–41.7	1.3	–20.4	4.8
M2	MeCN	Citrate-buffer ^2^	91.6	0.9	99.2	1.2	–41.2	7.8	–22.9	7.4
M3	MeCN/EA ^3^	Unbuffered	102.2	4.6	91.2	6.2	–44.2	5.9	–22.6	3.3
M4	MeCN/EA	Citrate-buffer	100.4	6.8	94.5	7.4	–47.9	14.6	–33.0	12.5
M5	EA	Unbuffered	95.4	18.3	91.1	8.7	–50.5	24.5	–34.6	17.1
M6	EA	Citrate-buffer	83.8	26.9	76.4	20.6	–45.1	30.1	–29.5	20.2

^1^ Original: 4g of MgSO_4_ and 1 g of NaCl. ^2^ EN 15662: 4 g of MgSO_4_, 1 g of NaCl, 1 g of Na_3_Citr·2H_2_O, and 0.5 g of Na_2_HCitr·1.5H_2_O. ^3^ MeCN/EA (1:1, *v*/*v*).

**Table 3:** Recovery rate and RSD analyzed after preparation using various purification methods.

Method	PurificationMethod	Recovery				Matrix Effect			
		Bifenthrin		Butachlor		Bifenthrin		Butachlor	
		Value (%)	RSD (%)	Value (%)	RSD (%)	Value (%)	RSD (%)	Value (%)	RSD (%)
M1	Untreated	93.7	4.7	100.0	0.2	–41.7	1.3	–20.4	4.8
M3	Untreated	102.2	4.6	91.2	6.2	–44.2	5.9	–22.6	3.3
M1-A	PSA ^1^	98.2	3.5	104.0	4.2	–3.2	2.5	–16.5	3.0
M3-A	PSA	101.8	1.7	89.4	10.5	–20.9	1.4	–14.3	7.6
M1-B	PSA + GCB ^2^	99.3	5.1	99.7	3.9	–9.0	1.1	1.9	3.2
M3-B	PSA + GCB	96.2	4.5	90.1	6.1	–7.6	3.2	–1.5	4.6
M1-C	HLB ^3^	102.6	3.5	103.5	2.9	–31.8	3.4	–24.2	4.6
M3-C	HLB	109.9	3.5	93.3	4.1	–32.3	1.3	–23.7	7.4
M1-D	PSA + GCB/HLB ^4^	100.5	5.0	107.1	6.2	–20.4	2.6	–16.3	5.9
M3-D	PSA + GCB/HLB	109.7	5.2	92.6	4.5	–29.2	1.1	–21.5	11.0

^1^ Primary secondary amine d-SPE: 25 mg of PSA and 150 mg of MgSO_4_. ^2^ Graphitized carbon black d-SPE: 25 mg of PSA, 7.5 mg of bulk carbonate, and 150 mg of MgSO_4_. ^3^ Oasis PRiME hydrophilic-lipophilic balance cartridge plus light (100 mg). ^4^ PSA + GCB d-SPE followed by PRiME HLB.

**Table 4:** The accuracy and precision of bifenthrin and butachlor for recovery and storage stability studies using the established method.

Pesticide	Study	Concentration (mg/kg)	Storage Period (days)	Accuracy (%)	RSD (*n* = 3) (%)
Bifenthrin	Recovery	0.01	-	101.3	1.8
		0.1	-	99.5	5.9
		2	-	88.0	4.0
	Storage stability	0.1	38	109.6	1.4
Butachlor	Recovery	0.01	-	107.3	2.6
		0.1	-	106.0	2.9
	Storage stability	0.1	25	104.3	4.9

**Table 5:** Evaluation of the matrix effects of bifenthrin and butachlor in chives from various sources and leafy vegetables (*n* = 3).

Crop	Sample	Source	Matrix Effect (% ME)			
			Bifenthrin		Butachlor	
			Value (%)	RSD (%)	Value (%)	RSD (%)
C1	Korean chives 1	Commercial market (used for method validation)	−5.2	3.2	−5.1	4.4
C2	Korean chives 2	Jeonju-si (harvested in the study)	−7.2	0.7	2.8	1.4
C3	Korean chives 3	Jincheon-gun (harvested in the study)	−18.8	0.7	2.1	5.2
C4	Chinese chives	Commercial market	−0.1	4.7	3.0	3.1
Sh	Shallot	Commercial market	4.2	0.7	1.9	3.8
Sp	Spinach	Commercial market	−11.0	1.9	7.2	0.9

**Table 6:** The residual results of the active ingredient for the bifenthrin 1 EC in chives.

Pesticide	Treatment Group	Pesticide Spraying Days Before Harvest	Concentration (mg/kg)			Average ± SD ^1^ (mg/kg)
			Rep. 1	Rep. 2	Rep. 3	
Bifenthrin	T1	7-0	0.971	1.025	1.010	1.002 ± 0.028
	T2	14-7	0.279	0.270	0.241	0.263 ± 0.020
	T3	21-14	0.122	0.126	0.124	0.124 ± 0.002
	T4	30-21	0.085	0.091	0.087	0.087 ± 0.003

^1^ Standard deviation.

## Data Availability

Data will be made available on request.
